# Comment on “Predictors of Echocardiographic Abnormalities in Asymptomatic Individuals With Increased Cardiothoracic Ratio Detected on Chest Radiography”

**DOI:** 10.1016/j.ahjo.2026.100982

**Published:** 2026-10-21

**Authors:** A. Aashish, P.B. Surendar, P. Prakash, D.M. Mahalakshmi

**Affiliations:** a Cardiology, Meenakshi Medical College Hospital & Research Institute, Meenakshi Academy of Higher Education and Research, Enathur, Kanchipuram, Tamil Nadu, 631552, India; b Arulmigu Meenakshi College of Nursing, Meenakshi Academy of Higher Education and Research, India; c Meenakshi College of Allied Health Sciences, Meenakshi Medical College Hospital & Research Institute, Meenakshi Academy of Higher Education and Research, India

**Keywords:** Cardiothoracic ratio, Echocardiography, Chest radiography, Cardiomegaly, Cardiac abnormalities, Asymptomatic individuals

Dear Editor,

Wattanawongwon et al. [1] are raising a question in settings where echocardiography is not widely available: which asymptomatic individuals with an increased cardiothoracic ratio actually need a scan? The study is grounded in concerns and the Thai setting gives it real-world relevance.. There is a deeper issue that goes beyond the limitations mentioned.

Echocardiography was performed at the discretion of the attending cardiologist. The methods say the decision was based on history, physical findings and ECG abnormalities.. These are the same factors the study then tries to analyze as predictors [2]. People were included in the study partly because a cardiologist already thought they were at risk—due to age, hypertension or an abnormal ECG. That means the sample was selected based on the factors the model is meant to assess [3]. What results is not a test of risk factors but a reflection of current referral practices. The paper does note that this selection may lead to a prevalence. But it doesn't fully address the problem: the odds ratios are likely biased in unpredictable ways. The recommendation to use age, hypertension and ECG-detected left hypertrophy to guide referrals ends up just echoing the existing system that created the sample. To properly answer the question the study would need a cohort where echocardiography was done regardless of judgment.

The composite outcome adds to the confusion. Abnormalities were found in 52.7% of patients. Most were not clinically significant. Left ventricular systolic dysfunction was seen in 1.5%, right ventricular dysfunction in 0.9% and moderate-to-severe valve disease in 4.6%. Left dilatation alone made up 41.8% of the total. So the composite is mostly a measure of atrial enlargement [4].. The predictors—age and hypertension—are well known to be linked with atrial enlargement. The discussion acknowledges that most findings were related to the atrium.. The abstract and conclusion still present the composite outcome as if half the cohort had meaningful structural disease. Reporting predictors separately for the important conditions—even with wide confidence intervals—would be far more helpful to a clinician trying to decide who to refer.

There are also three errors that need correction. The median estimated glomerular filtration rate is listed as 31.0 with a range of 33.0 to 52.0. That's impossible—the median cannot fall outside the range. The multivariable estimate for a history of stroke has a confidence interval from 0.66 to 0.35 which is backwards—the upper limit is lower than the lower limit.. Although backward stepwise selection is described, obesity, stroke history and systolic murmur are all in the final model despite *p* values of 0.06, 0.32 and 0.24. That suggests the selection rule used was not backward stepwise. The actual method applied should be clearly stated.

Despite these issues the underlying observation is important and well supported: a cardiothoracic ratio above 0.6 showed no association with abnormality. Mild enlargement on its own carries information. That finding, which challenges the use of cardiomegaly as a trigger, for referral is the most valuable part of the paper.. It stands independent of the statistical model.

## CRediT authorship contribution statement

**A. Aashish:** Conceptualization, Data curation, Formal analysis. **P.B. Surendar:** Visualization, Writing – original draft, Writing – review & editing. **P. Prakash:** Visualization, Writing – original draft, Writing – review & editing. **D.M. Mahalakshmi:** Visualization, Writing – original draft, Writing – review & editing.

## Informed consent

Not applicable.

## Ethical approval

Not applicable (Commentary on a previously published article).

## Declaration of Generative AI and AI-assisted technologies in the writing process

Generative AI tools were used only for language refinement and formatting assistance. All scientific interpretation, critical appraisal, and manuscript preparation were performed independently by the authors.

## Funding

None.

## Declaration of competing interest

The authors declare that there are no conflicts of interest.

## Data Availability

No new data were generated or analyzed in this study.
